# Cervical and Vaginal Deciduosis: Insights on Management and a Systematic Review of Observational Studies on Pregnancy Complications and Management Outcomes (Including Vaginal Birth)

**DOI:** 10.7759/cureus.44479

**Published:** 2023-08-31

**Authors:** Zoryana Bolgarina, Heet N Desai, Mithum Senaratne, Shivling S Swami, Soe Lwin Aye, Yash Trivedi, Abeer O Elshaikh

**Affiliations:** 1 Obstetrics & Gynecology, California Institute of Behavioral Neurosciences & Psychology, Fairfield, USA; 2 Internal Medicine, California Institute of Behavioral Neurosciences & Psychology, Fairfield, USA; 3 Internal Medicine, California Institute of Behavioral Neurosciences and Psychology, Fairfield, USA; 4 Internal Medicine/Family Medicine, California Institute of Behavioral Neurosciences & Psychology, Fairfield, USA

**Keywords:** antenatal bleeding, polypectomy, decidual polyp, decidual ectopy, systematic review

## Abstract

Introduction. Deciduosis is an ectopic transformation of connective tissue into decidual-like cells. This is the first systematic review describing the clinical course, associated pregnancy complications, and management outcomes of cervical and vaginal deciduosis.

Methods. Our search covered worldwide observational studies published in English in five databases (PubMed, PubMed Central (PMC), Europe PMC, ScienceDirect, and Google Scholar) from inception to February 24, 2023. We followed the Preferred Reporting Items for Systematic Reviews and Meta-Analysis (PRISMA) guidelines and critically appraised studies using CAse REport (CARE) and Joanna Briggs Institute (JBI) tools. Then, we extracted patient characteristics, clinical features, management-related information, and outcomes.

Results. The selection process identified 15 studies describing 30 pregnancies. Macroscopic cervical and vaginal deciduosis presented as recurrent vaginal bleeding in over 16 of 24 women (57%). Differential diagnoses included miscarriages, cervical pregnancy, placenta previa, and malignancy. Significant antenatal hemorrhages, preterm rupture of membranes, and preterm birth were the most frequent pregnancy complications. Only one of 27 electively performed procedures resulted in biopsy-induced uncontrolled vaginal bleeding (0.04%), suggesting the relative safety of the interventions. Lesion resection led to the cessation of recurrent symptoms in eight of eight patients (100%) compared to eight of 15 women (53%) under observation management. All women with polypoid deciduosis over 1.5 cm entered labor and delivered without complications.

Conclusions. We described the clinical course, pregnancy complications, diagnostic-related challenges, management, and associated outcomes in women with macroscopic cervical and vaginal deciduosis. We supported the analysis with the current state of the problem and discovered gaps for prospective studies.

## Introduction and background

Deciduosis, an extra-uterine transformation of connective tissue into decidual-resembling cells, mainly occurs during pregnancy. Microscopically, 70.2% of biopsies obtained during a cesarean section [1] and 15.2%-34% of cervical cytological smears [2,3], as well as up to 90% of cervical biopsies [4], revealed decidual cells. The incidence of macroscopic lesions is unknown.

Deciduosis is generally considered a benign reaction. However, it might lead to significant pregnancy complications and management challenges. Existing systematic reviews [5-7] listed deciduosis-associated complications, such as spontaneous hemoperitoneum in pregnancy, (peri)appendicitis, bowel perforation, endometriotic lesion decidualization, disruptions of such lesions (especially threatening when approximated to vulnerable areas like uterine vessels and ureters), urinary bladder or ovarian pseudotumor formation, hemothorax, catamenial pneumothorax, etc. However, to our best knowledge, no systematic review has analyzed deciduosis confined to the lower genital tract location and its impact on pregnancy.

Moving externally from the endocervix, deciduosis of the lower genital tract presents as polyp and ectopy, including its papillary, polypoid, and infiltrative forms [8]. Polyp and papillary ectopy arise from the stroma under the columnar epithelium and appear as multicolored "beans" and pale grape-like columnar epithelial villi enlargement, respectively. Polypoid ectopy occurs under the columnar and squamous epithelium, frequently includes a transformation zone, and presents as a friable, yellow-brown mass. Infiltrative ectopy originates under the squamous epithelium as multiple small elevations. Lesion ulceration is common.

Studies identified that pregnancy-associated changes in the cervix, including decidual cells or the Arias-Stella reaction, are sometimes mistakenly recognized as atypical [9,10]. Colposcopic impressions can be misleading while performing a biopsy during pregnancy might be risky because of the possibility of excessive bleeding and coincidental pregnancy complications [11]. Contrary to popular belief, it is most important to rule out the coexistence of malignancy in suspicious cases. 

Accumulated data suggest that cervical deciduosis increases the risks of late miscarriages and preterm birth because of a premature rupture of membranes [12-14]. Besides, large lesions in labor, especially those that lead to intrapartum bleeding, can raise additional challenges [15-16].

Therefore, this study aims to review the clinical course and management of pregnancies in patients with deciduosis of the lower genital tract. We have included all the possible pregnancy complications and analyzed the incidence of intervention-associated complications, symptoms, and lesion resolution according to the chosen management and, if lesions persisted, the size of the lesions and problems accompanying vaginal delivery. Summarizing these data, we describe the possible challenges, pregnancy complications, and management outcomes. Additionally, we discover knowledge gaps that might serve as a guide for subsequent report descriptions.

## Review

Methods

Search Strategy and Selection Process 

We followed the Preferred Reporting Items for Systematic Reviews and Meta-Analysis (PRISMA) guidelines released in 2020 to prepare a systematic review [17]. Our search strategy, using keywords such as deciduosis, ectopic decidua, ectopic decidual reaction, and ectopic decidualization, was introduced in five databases (PubMed, PubMed Central (PMC), Europe PMC, ScienceDirect, and Google Scholar) and retrieved the references from inception to February 24, 2023. Complete queries and the search results are provided in Table 1.

**Table 1:** The databases, search queries, and results The initial search showed: ^a^139 results on PubMed (applied filters: case reports, observational study); ^b^249 results on ScienceDirect (applied filters: case reports, research articles) PMC: PubMed Central

Databases	Queries	Results
PubMed	Deciduosis OR "Ectopic decidua" OR "Ectopic decidual reaction" OR "Ectopic decidualization" Filters: Case Reports, Observational Study	77^ a^
PMC	((Deciduosis) OR "Ectopic decidua") OR "Ectopic decidual reaction") OR "Ectopic decidualization"	159
Europe PMC	("Deciduosis" OR "Ectopic decidua" OR "Ectopic decidual reaction" OR "Ectopic decidualization")	215
ScienceDirect	"Deciduosis" OR "Ectopic decidua" OR "Ectopic decidual reaction" OR "Ectopic decidualization"	99^ b^
Google Scholar	"Deciduosis" OR "Ectopic decidua" OR "Ectopic decidual reaction" OR "Ectopic decidualization"	980

Following the search, we uploaded the citations into the EndNote software (Clarivate, Philadelphia, PA, USA) and shared them among the authors (ZB, HD, MS). Then, two reviewers (ZB and HD) independently proceeded with the identification and screening process. They were consulted by a third author (MS) whenever disagreements arose. After removing the duplicates, non-English, and irrelevant records, full-text articles were retrieved and reviewed for eligibility criteria, as provided in Table 2.

**Table 2:** Full eligibility criteria

Inclusion criteria	Exclusion criteria
1. Population: Pregnant women with lower genital tract deciduosis.	1. Associated ectopic or molar pregnancy
2. Phenomenon of interest: Clinical course of the pregnancies and unique difficulties	2. Type of studies: non-primary studies
3. Outcomes: Pregnancy complications and management-related outcomes	3. ‘Grey’ literature, including unpublished studies
4. Types of studies: Case reports, case series, and case-control studies	4. Animal studies
5. Human studies	
6. Time: Published from the inception to February 24, 2023.	
7. Location: Worldwide	
8. Language: Articles written in English	
9. Free full-text articles	

**Figure 1:**
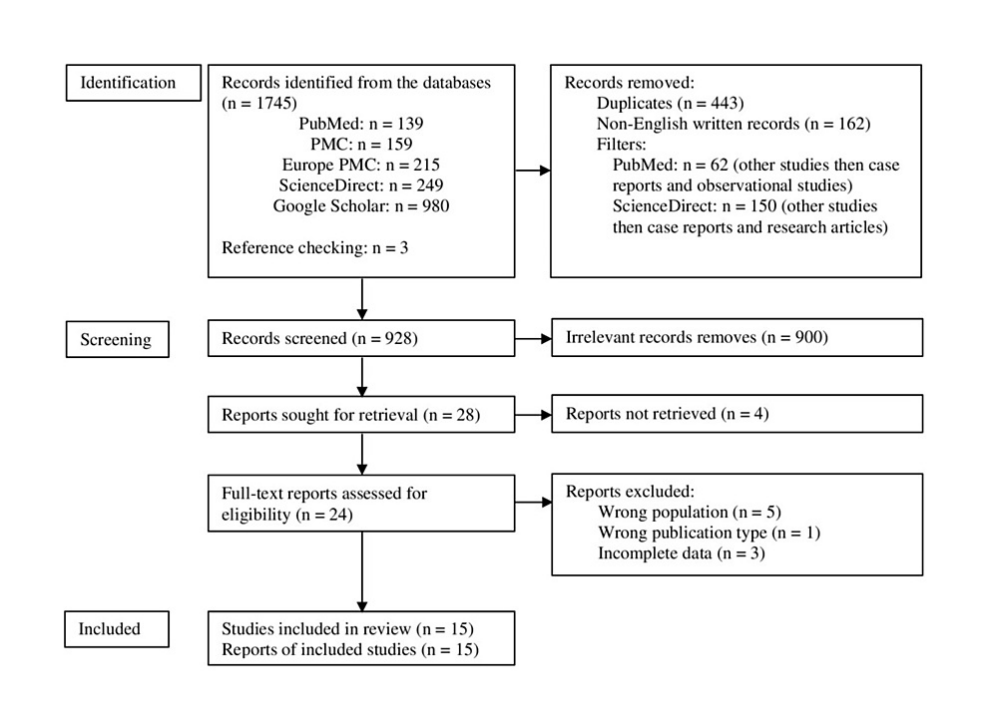
PRISMA flow diagram on the selection of studies PRISMA: Preferred Reporting Items for Systematic Reviews and Meta-Analysis; PMC: PubMed Central

Besides, checking reference lists retrieved three additional studies for the analysis. During the whole process, we did not use any automatic tools. The entire process is shown in Figure 1.

Data Collection and Synthesis

The same reviewers extracted data to a Microsoft Excel (Microsoft Corporation, Redmond, WA, USA) file independently according to the previously chosen variables and outcomes, which were as follows: (1) patients' characteristics included age, parity, preexisting history of cervical pathology and its treatment, and the results of cervical cancer screening; (2) clinical features included location, form, and size of the lower genital tract deciduosis, course of previous pregnancies, clinical presentation while establishing the diagnosis, and differential diagnosis; (3) management-related parameters: weeks of gestation (WG), results of the cytology and colposcopy and associated challenges, interventions (biopsy, polypectomy or removal of polypoid masses, or their combinations), and postpartum follow-up results within eight weeks; and (4) outcomes: (a) pregnancy complications, such as significant hemorrhages, pregnancy loss, intraamniotic infection (IAI) and/or preterm rupture of the membranes (PROM), preterm birth, cesarean section, etc.; and (b) management-associated results (intervention-associated complications, symptom resolution or persistence according to the chosen management, spontaneous regression of lesions during observation, and, if lesions persisted, their size and vaginal birth outcomes).

We made certain assumptions whenever information could not be recovered or clarified. If the authors reported multiple elevations on the cervix, we suspected an *infiltrative form* of deciduosis [18,19]. When the authors described the lesion as a single ectocervical or vaginal "mass," we interpreted it as a *polypoid form* of ectopy [20-22]. We recognized the case of having a decidual polyp during the previous pregnancy as a *polyp *[23]. In one case, it was hard to differentiate a polyp from a potentially papillary form of deciduosis or expulsed fragments of the decidua described as the endocervical "grayish membranes" [24]. If* vaginal bleeding* was not followed by any emergent workup, we considered it insignificant. Additional workup to rule out differential nosologies and concerns mentioned in the discussion sections of the articles contributed to the *differential diagnosis* analysis. We also recalculated the patient’s *age *based on the provided chronology [25] and the *term of gestation* based on the last menstrual period day or, if unavailable, the confinement date or size of uterus enlargement [22,23]. The *"reassuring" result of cytology* was interpreted as normal [19]. Given the diverse terms of gestation and circumstances of revealing the pathology, we *categorized pregnancies as managed expectantly* if: (1) there were no planned interventions [24,26]; (2) patients underwent urgent or emergent procedures [27,28]; (3) unprovoked pregnancy complications led to pregnancy termination [20,25]; or (4) patients presented initially after 37WG [15,16,21,23]. When reports included a scheduled remote postpartum follow-up for cervix re-evaluation within eight weeks, we suspected *lesion persistence* [19,22,28]. We excluded underreported and unrestored information from the corresponding analysis, marking them as "not reported" or "not applicable." The extracted data is organized in Tables 3-5. 

**Table 3:** Data extraction of the clinical characteristics NR: not reported; RV: rectovaginal; DIE: deep infiltrative endometriosis; VB: vaginal bleeding; CIN3: cervical intraepithelial neoplasia (grade 3); LLETZ: large loop excision of the transformation zone; Y: year(s); VD: vaginal discharge; PROM: preterm rupture of membranes; WG: weeks of gestation

Author, Year (Country)	Age	Parity	Preexisting cervical pathology/treatment and cancer screening	Location	Form (size)	Symptoms at the time of revealing the pathology	Preexisted pregnancy course	Differential diagnosis
Oh et al., 2022 (Korea) [20]	33	0	NR	Upper third of the vagina	Polypoid (RV DIE 6.1×4.1 cm)	Recurrent VB	Recurrent VB	Vaginal cancer, DIE malignization
Batkoska et al., 2022 (Slovenia) [25]	27	0	CIN3/LLETZ 5Y prior	Cervix	Polyp (2.2×1 cm)	VB	NR	None
	28	0	CIN3/LLETZ 6Y prior	Cervix	Polyp (4×1 cm)	Malodorous VD	NR	None
	29	0	CIN3/LLETZ 7Y prior	Cervix	Polyp (4×2 cm)	Resolved VB	NR	None
Verma et al., 2022 (India) [18]	28	2	NR	Cervix	Infiltrative (0.5-to-1.5 cm)	Resolved VB	NR	Cervical cancer
Buttery et al., 2021 (Australia) [16]	22	0	NR	Cervix	Polypoid (large)	NR	NR	Placenta previa
Mangla et al., 2021 (India) [27]	28	0	NR	Cervix	Polyp (2×2 cm)	Recurrent VB	Recurrent VB	None
Xiaoyin et al., 2018 (China) [26]	32	1	NR	Cervix	Polyp (0.8 cm)	VB	NR	NR
	25	0	NR	Cervix	Infiltrative/Polypoid (1 cm)	VB	NR	NR
	27	0	NR	Cervix	Ulcerated + Polyp	VB	NR	NR
	37	1	NR	Cervix	Polyp (2 cm)	Recurrent VD/VB	Recurrent VD/VB	NR
	28	0	NR	Cervix	Infiltrative/Polypoid (2 cm)	VB	NR	NR
	22	0	NR	Cervix	Papillary + Polyp	Recurrent VD/VB	Recurrent VD/VB	NR
	37	0	NR	Cervix	Polypoid (1.5 cm)	VD/VB	NR	NR
	29	0	NR	Cervix	Infiltrative + Polyp (2 cm)	Recurrent VB	Recurrent VB	NR
	27	0	NR	Cervix	Ulcerated/Polypoid (1.5 cm)	Recurrent VB	Recurrent VB	NR
	26	0	NR	Cervix	Polyp (1 cm)	VB	NR	NR
	23	0	NR	Cervix	Papillary/Polypoid (2 cm)	Recurrent VB	Recurrent VB	NR
van Diepen et al., 2015 (Netherlands) [19]	34	0	CIN3/LLETZ 1Y prior; Cytology: Normal 0.5Y and 1Y prior	Cervix	Infiltrative (0.5-to-1.5 cm)	VB	NR	Polyps, cervical adenoma
Oladipo et al., 2005 (UK) [28]	28	0	Insignificant history; Cytology: Normal 1Y prior	Cervix	Polypoid (4×4 cm)	VB of “300 ml”	“Uneventful”	Placenta previa or abruption
Gornall et al., 2000 (UK) [15]	23	0	Cytology: Normal 2Y prior	Cervix	Polypoid (8 cm)	PROM	“Offensive” VD	Cervical cancer
Armenia et al., 1964 (USA) [23]	22	2	Unremarkable cervix at 24WG	Cervix	Polypoid	None	Unremarkable	Primary reticulum cell sarcoma
	22	3	Previous pregnancy: 1-cm decidual polyp	Cervix	Polyp (6 cm)	VB	VB	“Undifferentiated tumor of reticuloendothelial origin”
Orr et al., 1961 (Northern Ireland) [29]	26	1	“Erosion”/Diathermy	Cervix + Upper third of the vagina	Polypoid (large)	Recurrent VB	Recurrent VB	Placenta previa, carcinoma
Mathie, 1957 (UK) [30]	39	2	NR	Upper third of the vagina	Polypoid (large)	None	“Uneventful”	Cervical, then vaginal carcinoma
Spivack, 1949 (USA) [24]	31	1	NR	Cervix	Infiltrative + "grayish membranes" (1.5 cm)	Worsened leukorrhea	Leukorrhea	Miscarriage, ectopic pregnancy
Lapan, 1949 (USA) [22]	33	0	“Normal”	Cervix + Upper third of the vagina	Polypoid (large)	Profuse VB	VB worsening	Cervical cancer
	35	1	NR	Upper third of the vagina	Polypoid (large)	None	NR	Vaginal cancer
	31	1	“Normal”	Cervix	Polypoid (large)	None	NR	Anaplastic cancer
Klein et al., 1946 (USA) [21]	23	0	12WG: No polyps, "erosions", or growths	Cervix	Polypoid (2 cm)	VB (“cupful”)	"Uneventful"	Placenta previa, cervical cancer

**Table 4:** Data extraction of the management results WG: weeks of gestation; NR: not reported; N/A: not applicable; VB: vaginal bleeding; NILM: negative for intraepithelial lesion or malignancy; cm: centimeter(s) ^a^The column "WG" refers to the initial revealing of pathology. The term of gestation for delayed or additional procedures was mentioned additionally in the corresponding fields. ^b^The term polypectomy included polypectomy and/or removal of the ectopic mass. ^c^The term pseudobiopsy means gentle tissue removal without obtaining a baseline layer.

Author, Year (Country)	WG^a^	Cytology	Colposcopy	Biopsy(ies)	Ectomy of lesions	Postpartum follow-up within 8 weeks
Oh et al., 2022 (Korea) [20]	34WG	NR	NR	N/A (Active VB)	None	NR
Batkoska et al., 2022 (Slovenia) [25]	6WG	NR	Polyp	None	Polypectomy	N/A (Removed)
	10WG	NR	NR	None	None	N/A (Removed vs. Regressed)
	7WG	NR	NR	None	Polypectomy	N/A (Removed)
Verma et al., 2022 (India) [18]	32WG	NR	NR	Biopsy	None	Speculum: Normal; Cytology: NILM
Buttery et al., 2021 (Australia) [16]	41WG	NR	NR	None (Intraoperative)	None	NR
Mangla et al., 2021 (India) [27]	20WG	NR	NR	None	Urgent polypectomy at 24WG	N/A (Removed)
Xiaoyin et al., 2018 (China) [26]	8WG	NILM	Polyp	None	None	N/A (Regressed)
	21WG	NILM	Ectopy	Biopsy	Polypectomy^b^ at 24WG	N/A (Removed)
	19WG	NILM	Polyp/Ectopy	Biopsy	None	N/A (Regressed)
	10WG	NILM	Polyp	Biopsy	Polypectomy at 12WG	N/A (Removed)
	15WG	NILM	Ectopy	Biopsy	None	NR
	16WG	NILM	Polyp/Ectopy	Biopsy	Polypectomy^b^ at 19WG	N/A (Removed)
	7WG	NILM	Ectopy	Biopsy	Polypectomy^b^ at 12WG	N/A (Removed)
	23WG	NILM	Polyp/Ectopy	Biopsy	Polypectomy^b^ at 27WG	N/A (Removed)
	32WG	NILM	Ectopy	Biopsy	None	NR
	8WG	NILM	Polyp	None	None	N/A (Regressed)
	28WG	NILM	Ectopy	Biopsy	Polypectomy^b^ at 31WG	N/A (Removed)
van Diepen et al., 2015 (Netherlands) [19]	11WG	Normal	Acetic acid: "no abnormalities"	Colposcopy-guided biopsy at 25WG	None	Speculum: Almost resolved; Cytology: Normal
Oladipo et al., 2005 (UK) [28]	28WG	NR	Unsatisfactory Acetic acid: “grayish-white”, fine punctuations, "atypical vessels"	Colposcopy-guided, multiple biopsies (Urgent settings)	None	Colposcopy: Normal
Gornall et al., 2000 (UK) [15]	38WG	NR	NR	Biopsies	None	Speculum: Normal; Colposcopy: Normal
Armenia et al., 1964 (USA) [23]	39WG	NR	N/A (Old study)	NR	None	Hysterectomy: 0.1×0.8 cm nodule
	15WG	NR	N/A (Old study)	Biopsy; Biopsies at 17WG	None	Cold knife conization: normal
Orr et al., 1961 (Northern Ireland) [29]	36WG	NR	N/A (Old study)	Biopsy	None	Speculum: Almost resolved
Mathie, 1957 (UK) [30]	28WG	NR	N/A (Old study)	Biopsy	None	Speculum: Normal
Spivack, 1949 (USA) [24]	10WG	NR	N/A (Old study)	None (pseudobiopsy^c^)	None	Speculum: "Inflamed and eroded”
Lapan, 1949 (USA) [22]	12WG	NR	N/A (Old study)	Biopsy	None	Speculum: "Erosion”; Biopsy: Diffuse adenomatosis
	12WG	NR	N/A (Old study)	Biopsy	None	Speculum: Normal
	˂12WG	NR	N/A (Old study)	Biopsy	None	Speculum: Normal
Klein et al., 1946 (USA) [21]	41WG	NR	N/A (Old study)	None	None	Speculum: “Erosion”; Biopsy: Normal

**Table 5:** Data extraction of the outcomes VB: vaginal bleeding; N/A: not applicable; C-section: caesarean section; IAI: intraamniotic infection; PROM: preterm rupture of membranes; UTI: urinary tract infection(s); NR: not reported; WG: weeks of gestation; VD: vaginal discharge; cm: centimeter(s); IOL: induction of labor ^a^The term polypectomy included polypectomy and/or removal of the ectopic mass. ^b^The term pseudobiopsy means gentle tissue removal without obtaining a baseline layer.

Author, Year (Country)	Pregnancy complications	Pregnancy course and immediate postpartum period			Vaginal birth: delivered/complication (peripartum size of the lesions)
		Planned procedure: Uneventful/Complication	Observation (including biopsy)/Polypectomy: Symptom(s) resolution or recurrence	Regression of lesions (if no ectomy was performed)	
Oh et al., 2022 (Korea) [20]	VB/Shock, Uterine scar, Preterm birth	N/A (Emergent settings)	Observation: Recurrent VB	N/A (Emergent C-section)	N/A (Emergent C-section)
Batkoska et al., 2022 (Slovenia) [25]	N/A (Remote missed abortion)	Polypectomy: Uneventful	Polypectomy: VB resolved	N/A (Removed)	N/A (Missed abortion)
	IAI/PROM, Late pregnancy loss	N/A (None)	Observation: Recurrent VB and UTI	N/A (Unknown: Removed vs. Regressed)	N/A (Late pregnancy loss)
	N/A (Polypectomy)	Polypectomy: Uneventful	Polypectomy: VB resolved	N/A (Removed)	N/A (Removed)
Verma et al., 2022 (India) [18]	Uterine scar, Preterm birth	Biopsy: Uncontrolled VB	Observation: NR	N/A (Urgent C-section)	N/A (Urgent C-section)
Buttery et al., 2021 (Australia) [16]	Uterine scar	N/A (Intraoperative)	Observation: NR	Persisted	Early stage of labor complicated by VB/fetal distress (entire posterior lip of the cervix)
Mangla et al., 2021 (India) [27]	VB/Shock	N/A (Urgent polypectomy)	Observation: Recurrent VB; Urgent polypectomy: NR	N/A (Removed)	N/A (Removed)
Xiaoyin et al., 2018 (China) [26]	NR	N/A (None)	Observation: NR	Regressed at 12WG	N/A (Regressed)
	NR	Biopsy: Uneventful; Polypectomy^a^: Uneventful	Polypectomy^a^: Recurrent VB resolution	N/A (Removed)	N/A (Removed)
	NR	Biopsy: Uneventful	Observation: Recurrent VB	Regressed at 35WG	N/A (Regressed)
	NR	Biopsy: Uneventful; Polypectomy: Uneventful	Polypectomy: Recurrent VB resolution	N/A (Removed)	N/A (Removed)
	NR	Biopsy: Uneventful	Observation: Recurrent VB	Persisted	NR
	NR	Biopsy: Uneventful; Polypectomy^a^: Uneventful	Polypectomy^a^: Recurrent VB resolution	N/A (Removed)	N/A (Removed)
	NR	Biopsy: Uneventful; Polypectomy^a^: Uneventful	Polypectomy^a^: Recurrent VB resolution	N/A (Removed)	N/A (Removed)
	NR	Biopsy: Uneventful; Polypectomy^a^: Uneventful	Polypectomy^a^: Recurrent VB resolution	N/A (Removed)	N/A (Removed)
	NR	Biopsy: Uneventful	Observation: Recurrent VB	Persisted	NR
	NR	N/A (None)	Observation: NR	Regressed at 20WG	N/A (Regressed)
	NR	Biopsy: Uneventful; Polypectomy^a^: Uneventful	Polypectomy^a^: Recurrent VB resolution	N/A (Removed)	N/A (Removed)
van Diepen et al., 2015 (Netherlands) [19]	N/A (C-section due to failure to progress)	Biopsy: Uneventful	Observation: NR	Persisted	N/A (Failure to progress)
Oladipo et al., 2005 (UK) [28]	Severe VB	N/A (Urgent settings)	Observation: "Without further complications"	Persisted	Delivered
Gornall et al., 2000 (UK) [15]	IAI/PROM, Uterine scar	Biopsies: Uneventful	Observation: “Offensive” VD	Persisted	N/A (Emergent C-section)
Armenia et al., 1964 (USA) [23]	None	NR	Observation: "Uneventful"	Persisted	Delivered (1.5×0.6×0.4 cm)
	None	15WG Biopsy: Uneventful; 17WG Biopsies: Uneventful	Observation: NR	Persisted	Delivered (2 cm)
Orr et al., 1961 (Northern Ireland) [29]	N/A (None)	Biopsies: Uneventful	Observation: Recurrent VB	Persisted	Delivered (large)
Mathie, 1957 (UK) [30]	“Mild toxemia”	Biopsies: Uneventful	Observation: Provoked VB	Persisted	IOL followed by mild VB (large)
Spivack, 1949 (USA) [24]	N/A (None)	N/A (Pseudobiopsy^b^)	Observation: Recurrent VB/Persisted leucorrhea	NR	NR vs. Regressed?
Lapan, 1949 (USA) [22]	N/A (None)	Biopsies: Uneventful	Observation: Provoked profuse VB	Persisted	Delivered
	N/A (None)	Biopsies: Uneventful	Observation: "Uneventful"	NR	NR vs. Regressed?
	N/A (None)	Biopsies: Uneventful	Observation: NR	Persisted	Delivered
Klein et al., 1946 (USA) [21]	Profuse VB	Early postpartum biopsy: VB	Observation: "Uneventful"	Persisted	Delivered (2 cm)

After completing the data extraction, two reviewers (ZB and HD) critically appraised the studies independently by using the CAse REport (CARE) assessment tool for case reports [31] and the Joanna Briggs Institute (JBI) critical appraisal tools for case series [32]. The third reviewer (MS) resolved doubts and disagreements. The studies that scored 70% or higher contributed to our systematic review. This threshold was lowered to 60% for the studies published before 2013, as we did not want to lose clinically relevant information due to differences in the requirements of reporting cases.

We summarized clinically relevant qualitative variables by frequencies and presented them as percentages supported by a numerator and denominator. Continuous variables were presented as the mean and range in a normal data distribution and the median and interquartile range in a non-normal data distribution.

We did not develop the protocol and register the systematic review at the International Prospective Register of Systematic Reviews (PROSPERO) due to the short timeline for its completion. The protocol was substituted with data extraction tables in Microsoft Excel and subsequent analysis.

Results

Our search strategy identified 1745 records. After removing the duplicates (n = 443), foreign language articles (n = 162), and, partially, irrelevant reports whenever filters could be applied (n = 212), 928 records underwent screening. The inability to retrieve four reports limited our assessment for eligibility to only 24 records; of those, we excluded nine studies due to the wrong population (n = 5) [33-37], wrong publication type (n = 1) [8], and incompleteness of the presented data for the analysis (n = 3) [14,38,39]. Finally, 15 of 17 studies scored from 63% to 100% during the critical appraisal process and were subject to analysis [15,16,18-30]; 14 case reports, and one case series describing 30 cases.

Patients' Characteristics

The mean patient age was 28 years (± 4.86). Nulliparous women represented the majority - 18 of 30 women (60%). Among preexisting pathologies, there was a history of cervical intraepithelial neoplasia (grade 3) treated with loop electrosurgical excision of the transformation (four cases), cervical "erosion" treated with diathermy (one case), and recurrent decidual polyps (three cases).

Clinical Presentation

Lesions were localized at the cervix (83%, 25 of 30 reports), the upper third of the vagina (10%, three of 30 reports), or both locations (7%, two of 30 cases). Among the 22 reported cases of ectopies (73%), 40% accounted for polypoid (12 cases), 10% were infiltrative (three cases), and 23% were coexisting forms (seven cases).

Clinically, deciduosis presented an accidental finding only in four of 29 patients (14%). The rest of the patients complained of leukorrhea or infectious vaginal discharge (21%, six of 29 women) and/or vaginal bleeding, which was the chief complaint in 22 of 29 women (76%) and had a recurrent character in more than 16 of 24 patients (57%) based on the previous and subsequent course of the pregnancies.

Differential diagnoses included threatened abortion and ectopic pregnancy (one case), cervical adenoma (one patient), placenta previa (four cases), and cervical or vaginal malignancy (11 cases), including one patient with suspicious rectovaginal deep infiltrative endometriosis malignization.

Management

Deciduosis was revealed before 24WG in 18 of 30 pregnancies (60%). All reported cytological findings were normal. Only one study documented the details of colposcopy. According to that study, the possible challenges are incomplete visualization of the transformation zone, "grayish-white" epithelium after applying acetic acid, and "atypical vessels."

Eleven of the patients were managed expectantly (37%). The rest of the pregnant women underwent a total of 27 planned procedures: before 24WG (12 cases), during 24-to-33 6/7WG (seven cases), and after 34WG (eight cases). In the eight weeks postpartum, five of 12 patients (42%) had residual findings, including "erosions" and diffuse adenomatosis.

Outcomes

Studies reported the following complications of the pregnancies: significant antenatal bleeding (four studies), late abortion (one case), IAI and/or PROM (two cases), preterm birth (two cases), and operative delivery with uterine scar formation (four patients).

Among the 27 scheduled procedures, only one case of uncontrolled vaginal bleeding occurred at 32WG (0.04%).

Observation, including pregnancies managed expectantly or biopsied, was followed by recurrent symptoms (vaginal bleeding, urinary tract infections, vaginal discharge) or uneventfully (none, single, or provoked episode) in eight (53%) and seven (47%) women, respectively. Recurrent vaginal bleeding resolved in all eight cases (100%) of performed polyp or polypoid mass ectomies.

Spontaneous regression of lesions during observation happened in 20% (three of 16 patients) at 12WG, 20WG, and 35WG. All of these lesions were polyps, including one case of ulcerated ectopy.

Nine studies reported lesion persistence until vaginal birth. Almost all of these women had large polypoid lesions during their pregnancies. Among them, six women entered labor with the reported size of the lesions ranging from 1.5-2 cm to "large." None of the cases were complicated with significant intrapartum hemorrhage, although one study reported fetal distress of unknown etiology in the early stage of labor.

Discussion

Deciduosis of the lower genital tract was observed in women of 22 to 39 years of age, appearing on the cervix (83%) and/or vaginal fornices. Unfortunately, most case reports did not report the prior history of cervical or vaginal pathologies, limiting the opportunity to suggest the recurrence nature of the pathology, although decidual polyps demonstrated the tendency [23,25].

We noted that macroscopic cervix and/or vaginal fornice deciduosis was most commonly symptomatic, presenting as recurrent painless vaginal bleeding in more than half of the patients. Three pregnancies with lesions exceeding 2 cm in diameter were complicated by spontaneous antenatal hemorrhage of over 250 ml between 24WG and 34WG [20,27-28]. According to the literature, the lesions start regression around 25WG and, in 60%-70%, disappear completely by 38WG [8]. Because of this, it seems reasonable to diagnose the condition early, while ruling out cancer and locating the placenta, and consider respiratory distress syndrome prevention, especially if a biopsy is unavoidable during the late term of gestation.

Reviewing a previous history of cervical dysplasia, its treatment, and subsequent surveillance, including cervical cytology obtained in the first trimester of pregnancy, might help establish the diagnosis while minimizing the risks associated with cervical biopsy. For example, in one study [19], a patient underwent treatment for cervical intraepithelial neoplasia (grade 3) with subsequent normal cytological results before conception. She developed an infiltrative form of deciduosis, followed by reassuring cytological plus colposcopy surveillance until 25WG when the biopsy was performed because of the growth of the lesions. This case highlights the importance of cervical cancer screening before 20WG [40] when the lesions do not obscure the transformation zone and awareness of the pathology (in this case, the infiltrative form does not include the transformation zone and is multifocal form, therefore can allow avoiding a biopsy). Moreover, the absence of a high-grade pattern, surrounding foci of lower-grade abnormalities, and intensive necrosis of the lesions during colposcopy might help to avoid biopsy if local guidelines do not require it, regardless of the dense whitening of the lesions. However, it is the polypoid form that seems to be the most problematic one from a differential perspective and the most reported form of ectopy (58%), as it includes a transformation zone. In addition to taking a history and performing the mentioned diagnostics, this form of deciduosis presents as confusingly friable compared to relatively solid cancer. The less frequently reported form of ectopy was the papillary one, which may be the less recognizable form of deciduosis, as likely shown in an example [24]. Still, in this case, expulsed fragments of the decidua are a suitable explanation too.

Previous studies noted that performing a biopsy of the cervix during pregnancy is generally safe in terms of the occurrence of significant bleeding or pregnancy complications [40-42]. We obtained similar findings based on 27 electively performed procedures before 24WG (44%), during 24-to-33 6/7WG (26%), and after 34WG (30%). Our analysis also reflects the relative safety of biopsy decidual lesions that might be more prone to hemorrhages due to their friability, frequent association with chronic inflammation, and susceptibility to necrosis [24], especially with pregnancy progression [8]. For instance, the included studies reported severe antepartum hemorrhage [20,27,28], profuse bleeding provoked by gynecologic evaluation [21], and biopsy-induced uncontrolled bleeding [18] in women between 24-41 WG. These lead to two conclusions: (1) a cervical biopsy or stiff brush procedure is needed if there are any doubts regarding cervical cancer [11], and (2) respiratory distress prophylaxis may be considered if the procedure is planned after 24WG. Lately, remote studies have indicated that misleading results might lead to unnecessary procedures such as cervical conization during pregnancy [39], postpartum hysterectomy, and cold knife conization [23].

The authors cite that cervical and/or vaginal deciduosis does not require special pregnancy management unless complications develop. However, there are a few considerations. First, a retrospective cohort study of 550 pregnant women with cervical polyps noted that 45.45% of cases accounted for decidualized ones, which led to a greater frequency of pregnancy complications than non-decidualized polyps (28.1% vs. 6.1%) [43]. Thus, managing decidualized polyps in certain circumstances can be distinct from those without changes. Second, decidual polyp or polypoid mass ectomies might be helpful in resolving recurrent symptoms, though it is uncertain who might benefit from the procedure and whether resection reduces the risk of pregnancy complications. Perhaps pregnant women over 11WG with a polyp width greater than 11 mm, recurrent vaginal bleeding (in our opinion, recurrent inflammatory discharges in the setting of either multicolored or fragile lesions), and visualized roots could be good candidates [25,44-46]. At the same time, one study noted that spontaneous regression of the lesions, which is common, did not reduce the risk of cervical insufficiency and pregnancy loss [13]. Again, the authors did not evaluate these outcomes based on the histological type of the polyps. Lastly, one case report showed a 6-to-2 cm polyp size reduction in two months on local treatment with Neosporin [23]. Although a natural regression process can explain it, similar options could be considered when the risk of symptomatic lesion removal is high (e.g., non-visualized roots) and infected and edematous lesions are suspected.

In labor, macroscopic polypoid lesions, especially large ones (e.g., covering the entire posterior lip or an 8-cm mass), are inflamed, and those that are initially revealed in the peripartum period concern providers regarding intrapartum bleeding and its differential diagnosis [15,16]. However, at least six old studies with the size of lesions between 1.5 and large cm were not complicated by significant intrapartum hemorrhage. In one report, the 2-cm polypoid lesion remained "silent" even in the early postpartum period until biopsy [21]. Finally, accelerated regression of cervical deciduosis is to be expected shortly after delivery and complete lesion resolution by six weeks after delivery. According to our analysis, some residual findings are possible.

Limitations

Our systematic review has a few limitations. There is a risk of publication bias associated with reporting only recognized and successfully managed cases. Of note, older studies reported mismanagement issues more openly. Although improved antenatal management and advanced diagnostic opportunities might explain it, the rarity of macroscopic lesions, the lesions' ability to appear at a later term of gestation, and uncertainty in managing some situations perplex providers [16,18]. We analyzed only studies published in English, which could lead to losing important information. Besides, some studies mainly described cases around the episode of active management, missing the information essential for a thorough evaluation and, consequently, limiting current knowledge on the diagnostic pitfalls that can be addressed in prospective case reports. We also recovered or clarified some missed and descriptive data (discussed in the Materials and Methods section) that unlikely impacted our results significantly. Of course, considering the rarity of the condition, we could include only studies with a lower grade of evidence. However, we covered an important segment useful for providers and prospective studies.

## Conclusions

We described the clinical course, all pregnancy complications, and management-associated outcomes in pregnant women with macroscopic cervical and/or vaginal deciduosis. Most frequently, it presented as recurrent vaginal bleeding episodes that can be substantial after 24WG, requiring the exclusion of placenta previa, cervical pregnancy, and malignancy, as well as consideration of respiratory distress syndrome prophylaxis (for instance, in cases of large lesions, planned procedures, and successfully treated hemorrhagic episodes). Pregnancy complications included significant antenatal hemorrhages, PROM, uncontrolled bleeding-associated late abortion or preterm birth, and operative delivery. Performing cervical cancer screening before 20WG, recognizing different forms of deciduosis, and confusing the friability of the lesions can minimize the risks related to performing a biopsy, which seems avoidable in most situations. However, a biopsy is necessary if there is any doubt regarding cancer (or guideline requirements), and it seems safe to biopsy decidualized lesions, especially before 24WG. Conservative management of the cases is commonly accepted, while the benefits and indications of the excision of the lesion require further evaluation. There are no reports of associated significant intrapartum hemorrhages in women with polypoid ectopies over 1.5 cm, which is a safe delivery route, although it might be individualized in some settings.

This review can serve as a standardized guideline for reporting all pertinent information in future case reports, filling the knowledge gap on diagnostic and management challenges in the context of current technological advancements and prenatal care standards. It can also be a quick source of information in the search for similar cases. There is a need to report cases of management concerning decidualized lesions in labor, regardless of their successful and complicated resolution. An interesting direction is investigating the debatable problem of managing cervical polyps in the presence or absence of decidualized changes.

## References

[1] Kim HJ, Kim MJ, Song JY, Kim MR, Kim YT (2008). The prevalence of deciduosis in fertile women during cesarean delivery. J Minim Invasive Gynecol.

[2] Ma L, Bian ML, Liu J, Wang XH, Pang CH, Chen Y (2011). Pregnancy related cervical cytological changes and clinical management. Chin J Obstet Gynecol.

[3] Schneider V, Barnes LA (1981). Ectopic decidual reaction of the uterine cervix: frequency and cytologic presentation. Acta Cytol.

[4] Laverty CR, Fortune DW, Davoren R, Butterfield LJ, Hollyock VE (1974). Stromal decidual change in the pregnant cervix: colposcopic, cytological and histological considerations. Aust N Z J Obstet Gynaecol.

[5] Masouridou S, Mamopoulos A, Mavromatidis G, Karagiannis V (2012). Endometriosis and perinatal outcome - a systematic review of the literature. Curr Womens Health Rev.

[6] Leone Roberti Maggiore U, Ferrero S, Mangili G (2016). A systematic review on endometriosis during pregnancy: diagnosis, misdiagnosis, complications and outcomes. Hum Reprod Update.

[7] Lier MC, Malik RF, Ket JC, Lambalk CB, Brosens IA, Mijatovic V (2017). Spontaneous hemoperitoneum in pregnancy (SHiP) and endometriosis - a systematic review of the recent literature. Eur J Obstet Gynecol Reprod Biol.

[8] Basta A (2006). Decidual ectopy of the uterine cervix. The Cervix.

[9] Pisharodi LR, Jovanoska S (1995). Spectrum of cytologic changes in pregnancy. A review of 100 abnormal cervicovaginal smears, with emphasis on diagnostic pitfalls. Acta Cytol.

[10] Michael CW, Esfahani FM (1997). Pregnancy-related changes: a retrospective review of 278 cervical smears. Diagn Cytopathol.

[11] Hunter MI, Monk BJ, Tewari KS (2008). Cervical neoplasia in pregnancy. Part 1: screening and management of preinvasive disease. Am J Obstet Gynecol.

[12] Nikolov A, Negentsov N, Maĭnkhard K, Mekhandzhiev Ts (2007). Course of pregnancy and delivery in cases with cervical deciduosis (Article in Bulgarian). Akush Ginekol (Sofiia).

[13] Hirayama E, Ebina Y, Kato K, Akabane-Nakagawa K, Okuyama K (2022). Cervical polyps in early pregnancy are a risk factor for late abortion and spontaneous preterm birth: A retrospective cohort study. Int J Gynaecol Obstet.

[14] McGee WB, Slate TA (1955). Decidual reaction of the cervix—a review of 27 cases. Calif Med.

[15] Gornall AS, Naftalin NJ, Brown LJ, Konje JC (2000). Massive necrosis of cervical ectopic decidua presenting in labour. BJOG.

[16] Buttery J, Harry A, Kapoor S (2021). Intrapartum hemorrhage secondary to circumferential ectopic cervical deciduosis: a case report. Case Rep Womens Health.

[17] Page MJ, Moher D, Bossuyt PM (2021). PRISMA 2020 explanation and elaboration: updated guidance and exemplars for reporting systematic reviews. BMJ.

[18] Verma ML, Sankhwar PL, Qayoom S, Gaur R (2022). Rare presentation of cervical deciduosis as antepartum haemorrhage. BMJ Case Rep.

[19] van Diepen DA, Hellebrekers B, van Haaften AM, Natté R (2015). Cervical deciduosis imitating dysplasia. BMJ Case Rep.

[20] Oh JW, Lee EJ, Jin YM (2022). Vaginal hemorrhage associated with decidualized rectovaginal deep infiltrating endometriosis during the third trimester of pregnancy: a case report. J Korean Soc Radiol.

[21] Klein J, Domeier LH (1946). An unusual decidual reaction in the cervix. Am J Obstet Gynecol.

[22] Lapan B (1949). Deciduosis of the cervix and vagina simulating carcinoma. Am J Obstet Gynecol.

[23] Armenia CS, Shaver DN, Modisher MW (1964). Decidual transformation of the cervical stroma simulating reticulum cell sarcoma. Am J Obstet Gynecol.

[24] Spivack M (1949). Shedding of decidua during uterine pregnancy and its clinical significance. Am J Obstet Gynecol.

[25] Batkoska M, Korošec S, Frangež HB (2022). A rare case of symptomatic recurrent decidual polyp in each pregnancy in a woman with primary infertility. Clin Exp Obstet Gynecol.

[26] Xiaoyin W, Qingping Z, Mei Y, HongYing Y (2018). Diagnosis and treatment of cervical ectopic decidua during pregnancy. Clin Exp Obstet Gynecol.

[27] Mangla M, Nautiyal R, Shirazi N, Pati B (2021). Ectopic cervical deciduosis: a rare cause of antepartum hemorrhage in mid trimester. Eurasian J Med.

[28] Oladipo A, Mathew J (2005). Cervicitis decidualis: a rare cause of antepartum hemorrhage. J Low Genit Tract Dis.

[29] Orr CJ, Pedlow PR (1961). Deciduosis of the cervix manifesting as antepartum hemorrhage and simulating carcinoma. Am J Obstet Gynecol.

[30] Mathie JG (1957). Vaginal deciduosis simulating carcinoma. J Obstet Gynaecol Br Emp.

[31] Gagnier JJ, Kienle G, Altman DG, Moher D, Sox H, Riley D (2013). The CARE guidelines: consensus-based clinical case reporting guideline development. Glob Adv Health Med.

[32] Munn Z, Barker TH, Moola S (2020). Methodological quality of case series studies: an introduction to the JBI critical appraisal tool. JBI Evid Synth.

[33] Richard F, Canlorbe G, Bazot M, Daraï E (2014). Management of pregnancy in woman with suspected malignant deep infiltrating endometriosis fistulised to the uterine cervix. BMJ Case Rep.

[34] Massi D, Susini T, Paglierani M, Salvadori A, Giannini A (1995). Pregnancy-associated ectopic decidua. Acta Obstet Gynecol Scand.

[35] Pafumi C, Pulvirenti G, Leanza V, Leanza G, Iemmola A, D’Agati A (2011). Severe dysplasia and spontaneous uterus rupture in labour. J Clin Res Bioeth.

[36] Exacoustos C, Lauriola I, Lazzeri L, De Felice G, Zupi E (2016). Complications during pregnancy and delivery in women with untreated rectovaginal deep infiltrating endometriosis. Fertil Steril.

[37] Kraemer B, Abele H, Hahn M, Wallwiener D, Rajab TK, Hornung R (2008). Cervical ectopic pregnancy on the portio: conservative case management and clinical review. Fertil Steril.

[38] Hilbert GH, Coleman FC (1951). Decidual polyps of the cervix. Am J Obstet Gynecol.

[39] Chapman GW, Savage EW, Salem FA (1979). Cervical deciduosis and intraepithelial neoplasia. J Natl Med Assoc.

[40] Economos K, Perez Veridiano N, Delke I, Collado ML, Tancer ML (1993). Abnormal cervical cytology in pregnancy: a 17-year experience. Obstet Gynecol.

[41] Basta A, Szczudrawa A, Pityński K, Kolawa W (2002). The value of colposcopy and computerised colposcopy in diagnosis and therapeutic management of CIN and early invasive cervical cancer in pregnant women (Article in Polish). Ginekol Pol.

[42] Baldauf JJ, Dreyfus M, Gao J, Ritter J, Philippe E (1996). Management of pregnant women with abnormal cervical smears. A series of 146 patients (Article in French). J Gynecol Obstet Biol Reprod (Paris).

[43] Zou J, He Y, Chen H, Wang P, Xiao X, Liu S (2022). A clinicopathologic analysis of decidual polyps: a potentially problematic diagnosis. Int J Clin Pract.

[44] Fukuta K, Yoneda S, Yoneda N (2020). Risk factors for spontaneous miscarriage above 12 weeks or premature delivery in patients undergoing cervical polypectomy during pregnancy. BMC Pregnancy Childbirth.

[45] Dadkhah F, Kashanian M, Eliasi G (2010). A comparison between the pregnancy outcome in women both with or without threatened abortion. Early Hum Dev.

[46] Takahashi Y, Iwagaki S, Itoh M (2017). Cohort study of the incidence of spontaneous preterm birth and septic abortion referred by pathological examination in Gifu prefecture in Japan. Early Hum Dev.

